# Watershed analysis in wedge resection of pulmonary pure ground-glass nodules hardly localized by CT-guided puncture

**DOI:** 10.1186/s12893-023-02034-2

**Published:** 2023-05-19

**Authors:** Zhilin Luo, Tianhu Wang

**Affiliations:** Department of Thoracic Surgery, The Third Affiliated Hospital of Chongqing Medical University, Chongqing, 401120 China

**Keywords:** Watershed analysis, Pulmonary pure ground-glass nodules, Wedge resection

## Abstract

**Background:**

To investigate the feasibility and safety of watershed analysis after target pulmonary vascular occlusion for the wedge resection in patients with non-palpable and non-localizable pure ground-glass nodules during uniport thoracoscopic surgery.

**Methods:**

A total of 30 patients with pure ground-glass nodules < 1 cm in diameter, localized in the lateral third of the lung parenchyma, were enrolled. Three-dimensional reconstruction of thin-section computed tomography (CT) data was performed using Mimics software before surgery to observe and identify the target pulmonary vessels supplying the lung tissue in the area where the pulmonary nodules were localized and to temporarily block the target pulmonary vessels during surgery. Next, the extent of the watershed was determined with the expansion-collapse method, and finally, wedge resection was performed. After wedge resection of the target lung tissue, the blocked pulmonary vessel was released, thus allowing operators to complete the procedure without damaging pulmonary vessels.

**Results:**

None of the patients experienced postoperative complications. The chest CT of all patients was reviewed six months after the operation, revealing no tumor recurrence.

**Conclusions:**

Our results suggest that watershed analysis following target pulmonary vascular occlusion for wedge resection in pulmonary pure ground-glass nodules is a safe and feasible approach.

## Background

The increasing popularity of chest computed tomography (CT) during physical examinations has resulted in an increasing number of detected pulmonary nodules [1]. Fleischner Society guidelines recommend surgical resection for these patients if the nodules are enlarged after regular follow-up observation [2]. According to the results of Study JCOG0804/WJOG4507L in Japan, segmentectomy or wedge resection is suitable for small pulmonary nodules ≤ 2 cm in diameter and consolidation tumor ratio (CTR) < 0.25 [3]. However, as a segmentectomy is difficult to perform, wedge resection may be offered for nodules localized in the outer third of the lung. However, for pure ground-glass nodules < 1 cm in diameter, localization is often required before surgery due to no solid component, palpating difficulty during the operation, and occasional difficulty in finding the resected specimen. At present, common methods for localization include preoperative CT-guided percutaneous localization and fluorescence thoracoscopy [4]. Yet, for some nodules localized at the apex of the lung, obstructed by the rib and scapula, or close to the great vessels of the heart, there are some difficulties in the preoperative placement of locating needles. Meanwhile, the purchase of equipment such as fluorescent thoracoscopy (about 3 million RMB) and electromagnetic navigation bronchoscopy (about 5 million RMB) requires high costs, and many hospitals do not have such equipment. Consequently, in order to solve the above problems, we conducted a watershed analysis method [5]. In this study, we investigate the feasibility and safety of watershed analysis following target pulmonary vascular occlusion for the wedge resection in patients with non-palpable and non-localizable pure ground-glass nodules during uniport thoracoscopic surgery.

## Methods

### Materials

The clinical data of 30 patients (13 males and 17 females, aged 41–78 years) with pulmonary pure ground-glass nodules who underwent surgery at our hospital between January 2020 and October 2021 were retrospectively collected. Inclusion criteria were as follows: (1) Any pure ground-glass nodule less than 1 cm in right or left upper lung was found under preoperative chest enhanced CT scan (256-slice CT scanner[General Electric Company], the slice thickness and interlayer spacing are both 0.625 mm. The window width and window level of the CT mediastinal window are 350 Hounsfield units (HU) and 35 HU, respectively.); (2) patients were regularly followed up for observation; with indications for operation identified after preoperative discussion, and wedge resection was proposed; (3) lesions were localized in the lateral third of the lung parenchyma, and were obstructed by the scapula or rib, or close to the great vessels of the heart, and were 7-9.5 mm in diameter. Exclusion criteria were: (1) patients with obvious contraindications to surgery, such as heart and lung function disorders; (2) Patients who are easy to puncture and locate under CT before surgery. Three-dimensional reconstruction with Mimics 21.0 software was used in all patients to determine the location of nodules, including 6 cases in RS1, 4 cases in RS2, 4 cases in RS3, 11 cases in LS1 + 2, and 5 cases in LS3.

All procedures were performed in accordance with the ethical standards laid down in the 1964 Declaration of Helsinki and its later amendments. The present study was approved by the Ethics Committee of the Third Affiliated Hospital of Chongqing Medical University. Informed consent was waived by the same committee that approved the study (the Third Affiliated Hospital of Chongqing Medical University) in the ethical approval and consent section.

### Typical procedures

#### Preoperative three-dimensional reconstruction

**Fig. 1:**
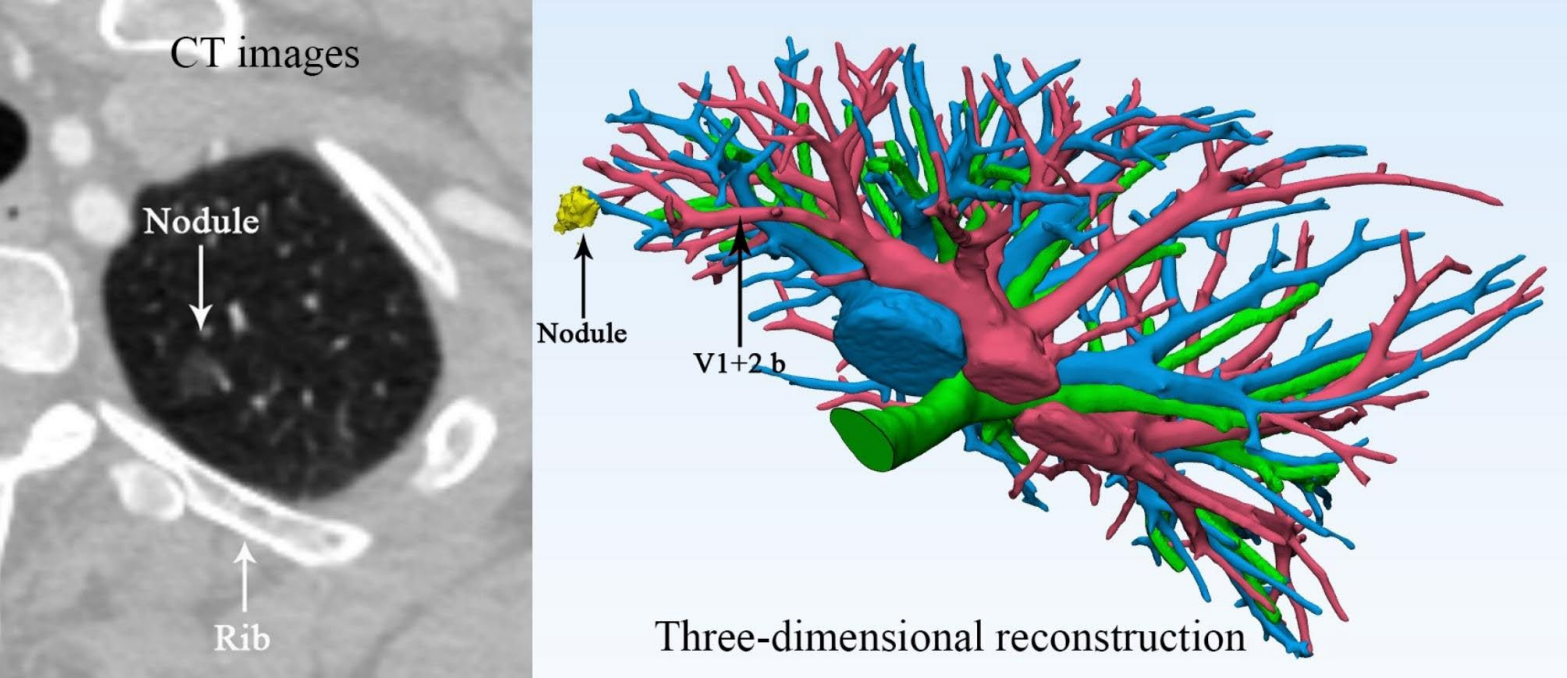
CT images of the plane with the largest diameter of the lesion and 3d reconstruction of pulmonary vessels of the patient

Mediastinal window enhanced CT data with a slice thickness of 0.625 mm were imported into Materialise Mimics 21.0 software (Belgium) for 3D reconstruction of nodules, trachea, and pulmonary vessels. The choice to import mediastinal window enhanced CT data instead of lung window data is based on its greater suitability for reconstructing pulmonary vessels. In Mimics software, when observing ground glass nodules, we can adjust the imported mediastinal window CT data to lung window data using the contrast adjustment function. The data were in DICOM format, and each 3D reconstruction takes approximately half an hour. During the reconstruction process, great efforts were made to reconstruct the peripheral pulmonary vessels to observe the pulmonary vessels closest to the nodules. The principles for selecting the pulmonary vessels to be blocked included: (1) pulmonary vessels closest to nodules; (2) it was not necessary to select the artery, but as long as it was easy to dissect and block under the uniport thoracoscopy, the vein was also considered as a good choice; (3) based on the size and location of the nodules, as well as the ease of dissection, the location for blocking the pulmonary vessels during surgery was at segment level or sub-segment level (Fig. 1).

#### Surgical procedures

**Fig. 2:**
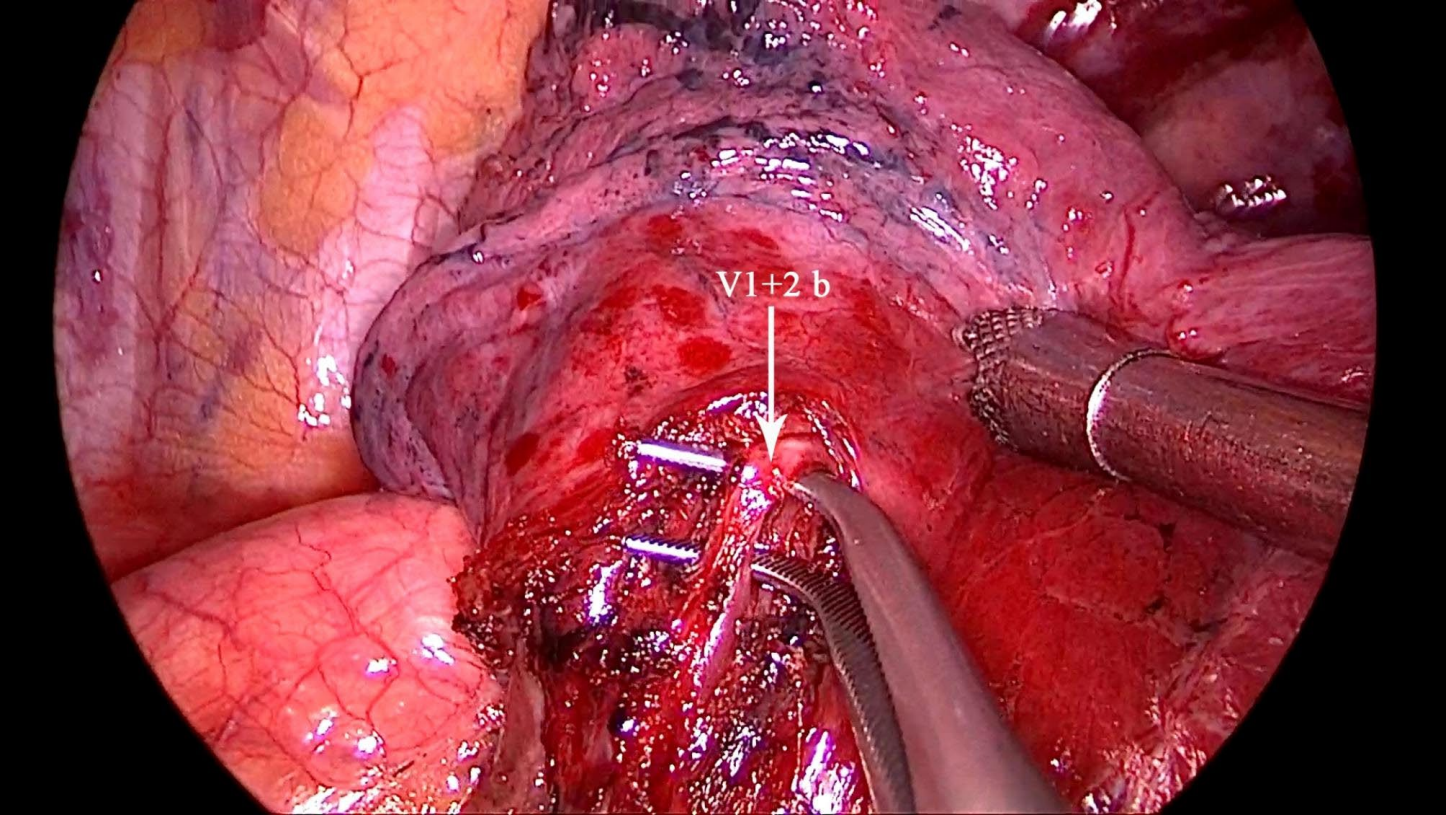
Dissecting the vessels to be blocked

**Fig. 3:**
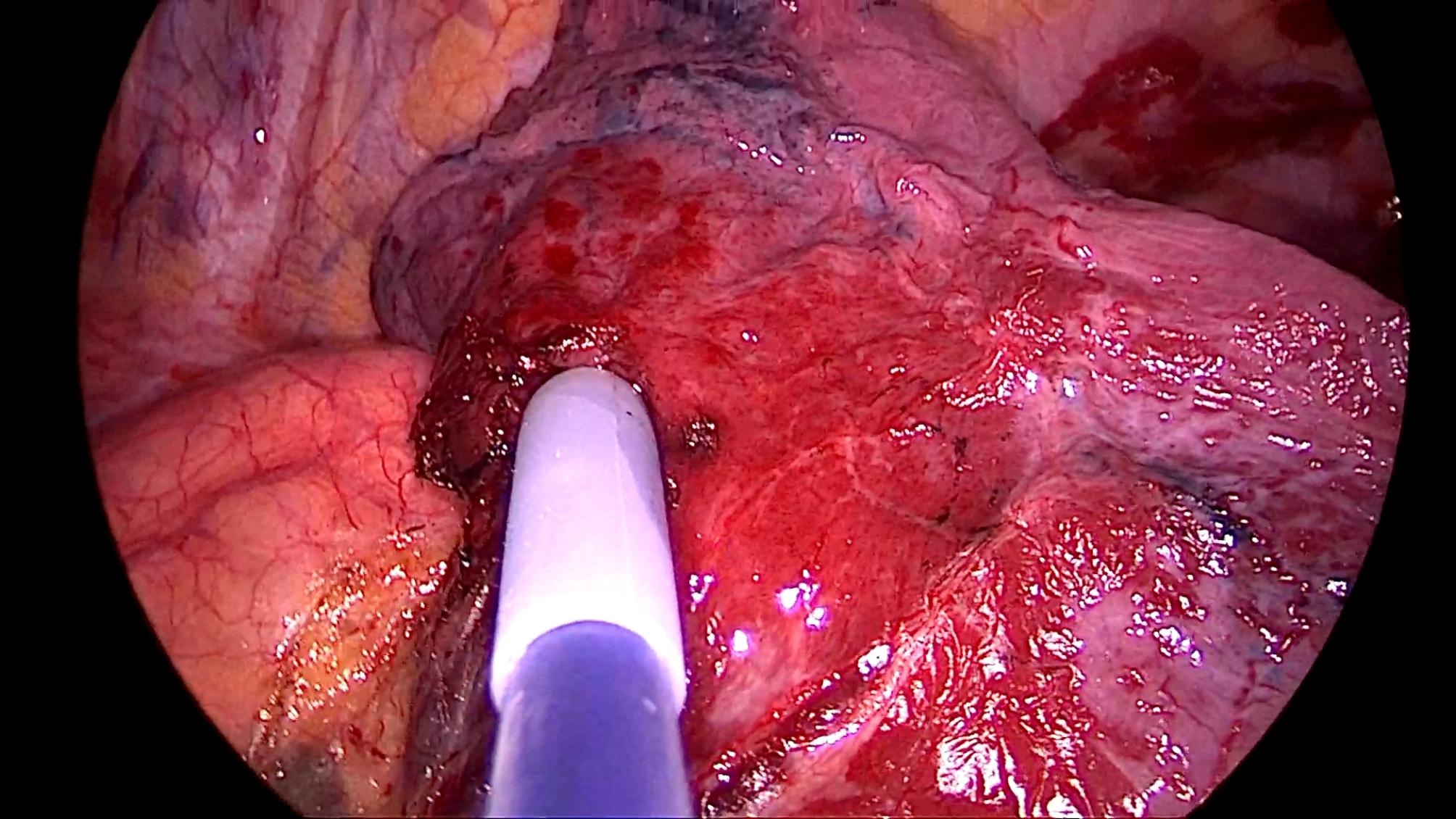
Block blood vessels

**Fig. 4:**
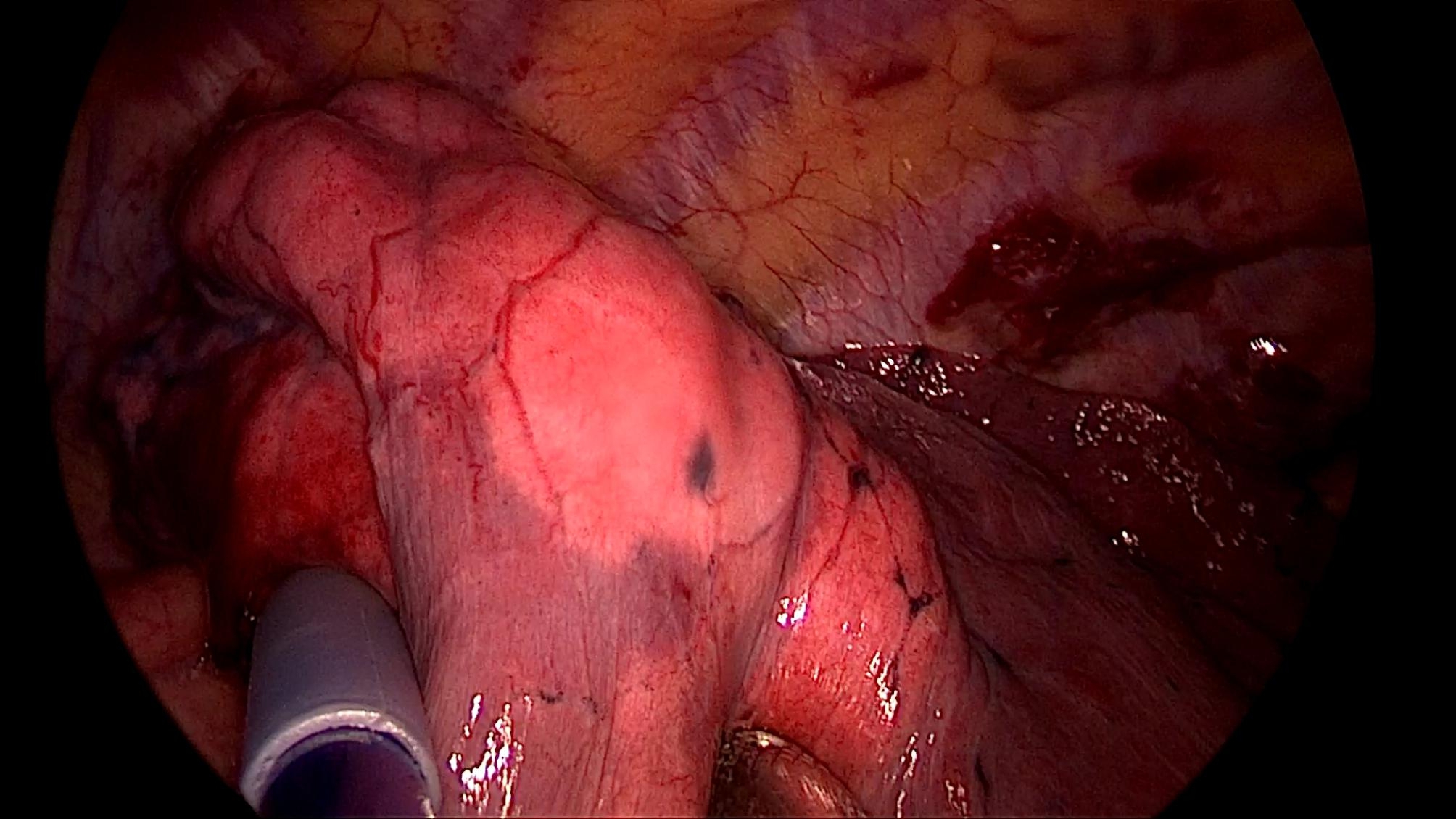
The lung tissue to be resected was shown by the distension-collapse method

**Fig. 5:**
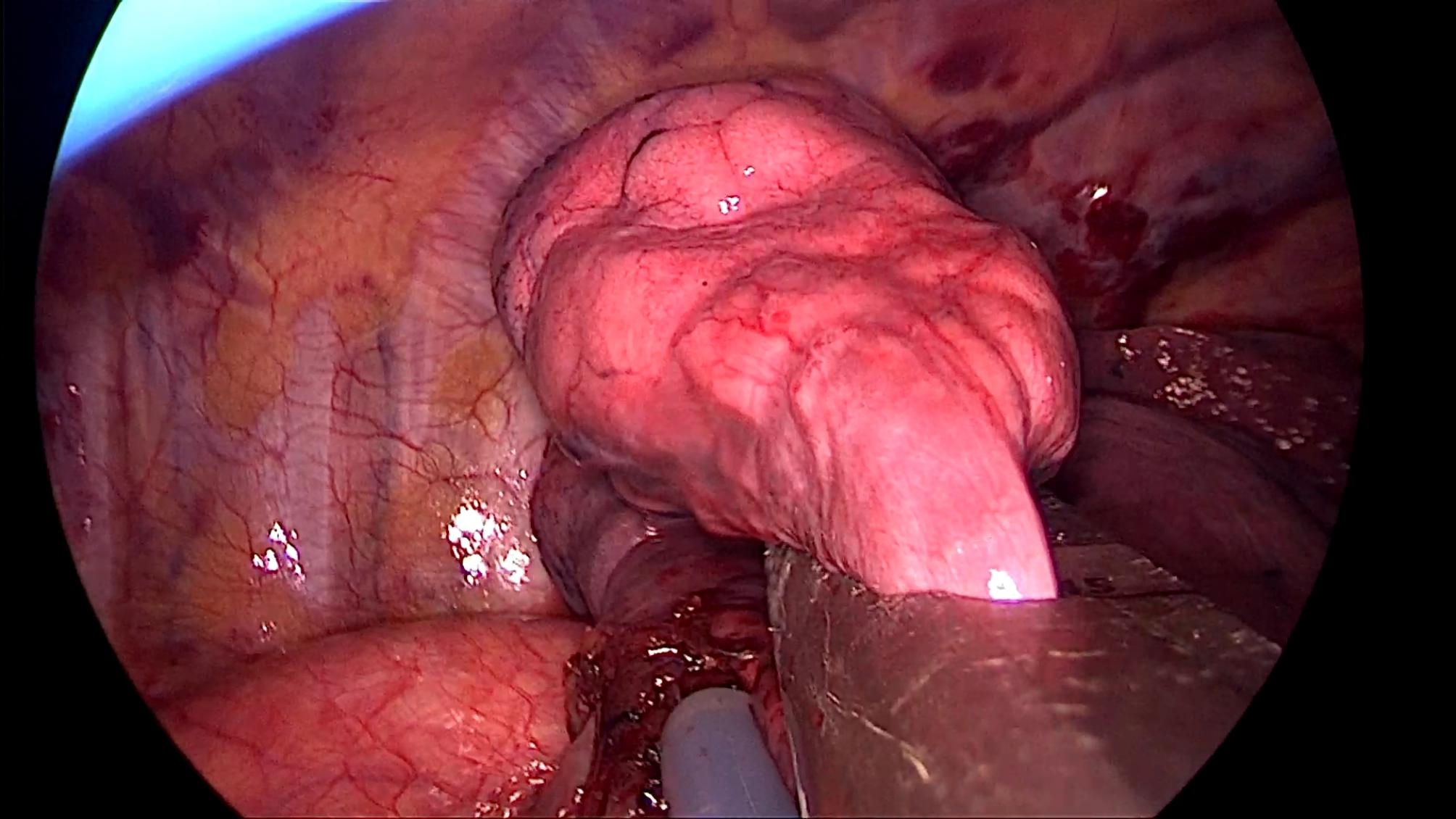
Excise the target lung tissue

**Fig. 6:**
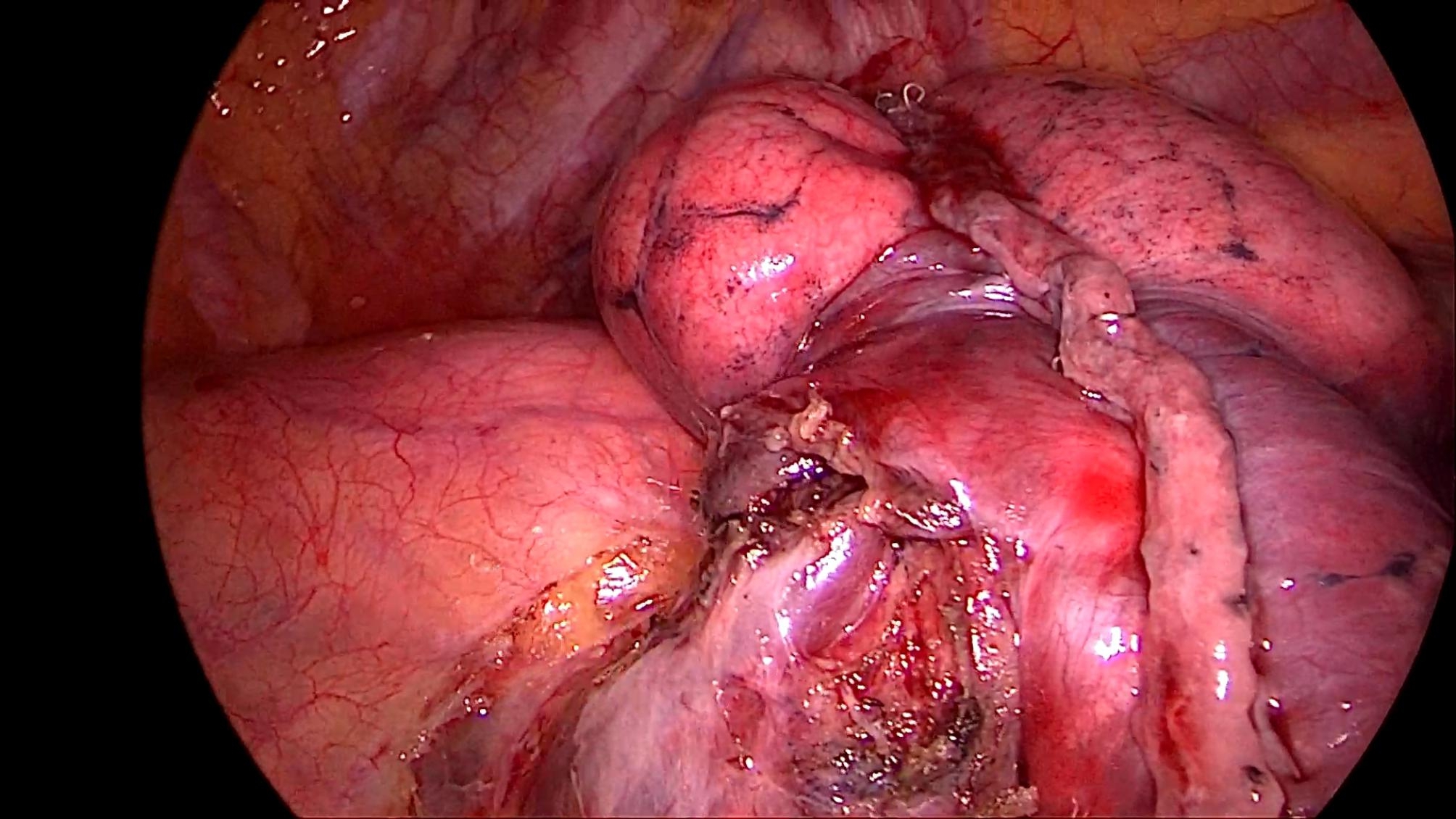
The disposable Rummel tourniquet was then released

**Fig. 7:**
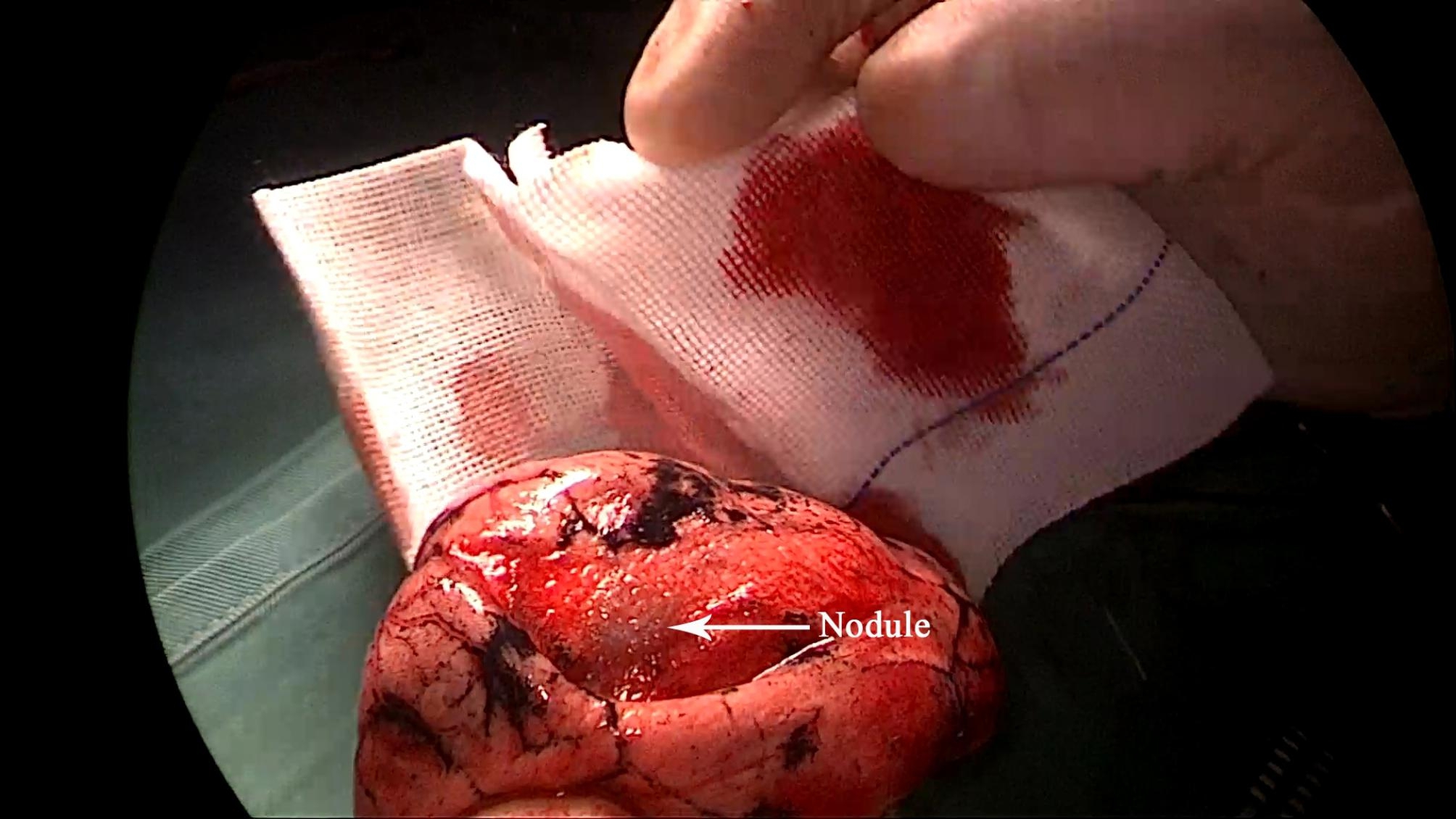
Find the lesion

All patients underwent the operation under a uniport thoracoscopy without preoperative localization, as one of the inclusion criteria for this study was patients with difficult localization. All cases were wedge resections, as using the method in this study is simpler than segmental resection. The surgical procedure is as follows.The anesthesia was performed under general anesthesia with double-lumen endotracheal intubation for single-lung ventilation, and the patient was positioned in lateral decubitus position on the healthy side during the operation. The chief surgeon and the thoracoscopic assistant stood to the ventral side of the patient. The incision was localized in the 4th intercostal space at the affected side, between the anterior axillary line and mid-axillary line, with a length of about 3 cm. After cutting open the chest wall muscles, an incision protective sleeve was placed. Then based on the preoperative three-dimensional reconstruction, by referring to the location of pulmonary nodules, the course of pulmonary artery and vein, and considering the nodule size, safe resection margin, and other factors, we identified the target pulmonary artery or pulmonary vein, which was supplying the lung tissue in the area of the localized pulmonary nodules, for intraoperative blocking. Later, the target pulmonary vessel to be blocked was dissociated during surgery (Fig. 2) and blocked with a disposable Rummel tourniquet(a device used to occlude blood flow during surgery) (Fig. 3). Next, based on the idea of the distension-collapse method [6], pure oxygen was used to expand the lung. After waiting several minutes, the lung tissue outside the watershed of the blocked pulmonary vessel collapsed while the lung tissue remained inflated [7, 8] (Fig. 4). Next, the targeted lung tissue was excised with a stapler along the distension-collapse boundary (Fig. 5). The disposable Rummel tourniquet was then released, and the blocked pulmonary vessel resumed normal function (Fig. 6). The remaining lung tissue was well re-expanded, and the lesion was eventfully found in the resected lung tissue (Fig. 7). Next, water was injected into the thoracic cavity, after which the anesthesiologist was instructed to expand the lung and to observe whether any air leakage occurred. Finally, a closed thoracic drainage tube was placed, and the chest part was closed layer by layer, thus completing the operation.

## Results

The patients’ characteristics are shown in Table 1. The operation was successfully completed, and the lesion was found in all patients. The distribution of arterial or venous blocked case numbers is shown in Table 2. Also, paraffin section examination of lung resection margin in all patients revealed no cancer cells, and measured with a ruler, the surgical margins of all nodules are greater than or equal to 2 centimeters (Table 3). The surgical duration was 73.4 ± 11.5 min, the amount of intra-operative bleeding was 30.7 ± 10.5 mL, the duration of postoperative drainage was 1.5 ± 0.6 d, the duration of postoperative hospital stay was 3.7 ± 0.9 d, and there was no death during the hospitalization (Table 3). None of the patients had postoperative complications, and all recovered uneventfully. Postoperative pathology revealed AAH in 6 cases, AIS in 13 cases, and MIA in 11 cases. The chest CT of all patients was reviewed six months after the operation, revealing no tumor recurrence and good lung expansion. Meanwhile, all patients are living well, keeping the same quality of life as their preoperative status.

**Table 1:** Demographic and clinical characteristics of patients (N = 30)

Variable	Number
Age (years)	57.8 ± 9.5
Male/Female	13/17
Lesions in diameter	All with a diameter of 7–9.5 mm
Lesions location	
RS1	6
RS2	4
RS3	4
LS1 + 2	11
LS3	5
Paraffin section pathology	
AAH	6
AIS	13
MIA	11

**Table 2:** Distribution of arterial or venous blocked case numbers

Variable	the number of arterial blocked patient	the number of venous blocked patient
**RS1**	**2**	**4**
**RS2**	**2**	**2**
**RS3**	**1**	**3**
**LS1 + 2**	**4**	**7**
**LS3**	**3**	**2**

**Table 3:** Operative data of patients (N = 30)

Variable	Measurement
Operation time(min)	73.4 ± 11.5
Estimated blood loss(ml)	30.7 ± 10.5
The range of the long diameter of all nodules(mm)	7-9.5
The surgical margins of all nodule (cm)	≥ 2
Postoperative drain duration(d)	1.5 ± 0.6
Postoperative hospital stays(d)	3.7 ± 0.9
Complications	None

## Discussion

For pulmonary nodules with a diameter smaller than 1 centimeter detected by CT examination, surgical treatment may be considered if the lesion increases in size or density during follow-up. In some cases, surgery can also be considered for patients who experience severe anxiety due to pulmonary lesions. Therefore,in this study, we presented an inexpensive, safe, and feasible method for patients with non-palpable pulmonary pure ground-glass nodules that are difficult to be localized by CT-guided puncture. Also, our approach did not cut off blood vessels in the lungs. With the preoperative three-dimensional reconstruction, the location of pulmonary nodules and the pulmonary vessels supplying the lung tissue around the pulmonary nodules were accurately identified, after which the pulmonary vessels were blocked during surgery so that the watershed range of blocked pulmonary vessels could be clearly displayed, creating conditions for accurate wedge resection. Following are the advantages of this approach: (1) it is a simple, economical method that does not require expensive equipment such as fluorescence thoracoscope and electromagnetic navigation bronchoscope; therefore, it can be applied in any hospital performing thoracoscopic surgery. (2) The proposed method is characteristically a wedge resection but without the need to expose and transect the bronchus, which makes it more simple than segmentectomy. Also, the blocked vessels are released after the operation without causing damage to the vessels, which makes the operation safer, and causes less trauma to the patient. (3) Safe margin is usually the most important issue in pulmonary wedge resection [9] that our method can successfully solve. The position of pulmonary nodules in the lung does not change, so as long as the preoperative three-dimensional reconstruction is correctly completed to allow for the identification of vessels supplying for the lung tissue around the pulmonary nodules, the watershed range of target pulmonary vessels can be determined by blocking the target pulmonary vessels and then using the inflation-collapse method [6] to ensure safe resection margin and ensure successful removal of the lesion without omission. (4) Our method is simple to operate. We only need to block the most easily dissected vessel from the perspective of surgical operation. As long as this vessel supplies the pulmonary tissue in the region where the pulmonary nodule is localized and is adjacent to the nodule, whether it is a vein or an artery, it can be blocked. However, there is a slight difference in the boundary displayed when blocking a vein compared to an artery due to the different distribution of their branching patterns. Nevertheless, this difference does not affect our surgical procedure, as long as the pulmonary nodule is localized within the lung tissue supplied by the blocked vessel (regardless of whether it is a vein or an artery), we can achieve our goal.In addition, in the process of dissecting and dissociating the target vessel, it is necessary to use an ultrasonic scalpel or electric coagulation hook to slightly separate the lung parenchyma around the root of the target vessel, during which postoperative air leakage may occur. Considering this matter, in the present study, we used an argon knife to treat the leaking lung tissue at the end of the operation and then covered the lung section with oxidized regenerated cellulose (Johnson & Johnson, USA) and porcine fibrin sealant (Guang Zhou Bioseal, China). Following such treatment, air leakage could be effectively prevented, and for all patients in this study, the postoperative extubation was performed after 1.5 ± 0.6 d. The time of air leakage and chest tube removal is similar to that of the traditional method.

## Conclusions

In conclusion, watershed analysis following blocking the target pulmonary vessels provides a safe, effective, and economical surgical procedure for the precise wedge resection in pulmonary pure ground-glass nodules, with adequate resection margins. Furthermore, this approach is suitable for patients with pulmonary pure ground-glass nodules non-localizable under CT guidance before the surgery due to obstruction by the scapula or rib or close position to the great vessels of the heart, which makes it worthy of being widely popularized in the clinical application.

## Data Availability

The datasets used and/or analyzed during the current study are available from the corresponding author on reasonable request.

## List of Abbreviations

CT: Computed tomography
CTR: Consolidation tumor ratio
